# Injuries prior and subsequent to index poisoning with medication among adolescents: a national study based on Norwegian patient registry

**DOI:** 10.1186/s12888-018-1778-8

**Published:** 2018-06-18

**Authors:** Ping Qin, Shihua Sun, Anne Seljenes Bøe, Barbara Stanley, Lars Mehlum

**Affiliations:** 1 National Centre for Suicide Research and Prevention, Institute of Clinical medicine, University of Oslo, Sognsvannsveien 21, N-0372 Oslo, Norway; 2 Department of Epidemiology, Shandong University School of Public Health and Shandong University Center for Suicide Prevention Research, Jinan, China; 3 Department of Psychiatry, Columbia University College of Physicians and Surgeons, New York, NY USA

**Keywords:** Self-injurious behaviour, Medication poisoning, Adolescence, Emergency health services, Population study

## Abstract

**Background:**

Adolescents treated for self-poisoning with medication have a high prevalence of mental health problems and constitute a high-risk population for self-harm repetition. However, little is known about whether this population is also prone to injuries of other forms.

**Methods:**

Data were extracted from the Norwegian Patient Registry to include all incidents of treated injuries in adolescents aged 10–19 years who were treated for self-poisoning with medication during 2008–2011. This longitudinal approach allowed for the inclusion of injuries of various forms both before and after the index poisoning with medication. Gender differences and associations of injuries with recorded deliberate self-harm or psychiatric comorbidity at index poisoning were analysed. Forms of injury and psychiatric illnesses were coded according to the ICD-10 system.

**Results:**

1497 adolescents treated for self-poisoning with medication were identified from the source database, including 1144 (76.4%) girls and 353 (23.6%) boys. For these 1497 adolescents a total of 2545 injury incidents were recorded in addition to the index poisoning incidents, consisting of 778 injury incidents taking place before the index poisoning and 1767 incidents taking place subsequently. Altogether 830 subjects (55.4%) had an injury treated either before or after the index poisoning. Injuries to the hand and wrist as well as injuries to the head, neck and throat were predominant in males. Females were more likely to repeat poisoning with medication, particularly those with psychiatric disorders.

**Conclusion:**

Adolescents treated for poisoning with medication represent a high-risk population prone to both prior and subsequent injuries of other forms, and should be assessed for suicidal intent and psychiatric illness.

## Background

Non-fatal injuries are the leading cause of disability among children and adolescents worldwide [1]. Although accidental injuries account for a large proportion of such incidents in the population under 18 years in most countries, self-inflicted injuries are also prevalent [1]. It is well-documented that adolescents with a history of self-poisoning with medication are at a high risk for repetition of self-poisoning and even death by suicide [2–8]. However, little is known to what extent these adolescents are prone to injuries of other types before and after the medication poisoning episode.

According to community studies [9–11], self-cutting is more prevalent than self-poisoning among adolescents. A switch of injury method is observed in individuals who repeatedly injure themselves [6, 12]. Multiple methods used in repeated injuries have been found to predict suicide attempts [13]. At the same time, there are gender differences in the incidence as well type of injuries among adolescents [14–17]. While male adolescents have higher overall rates of injuries of all types than their female peers [1, 18, 19], female adolescents show a higher incidence in self-inflicted injuries [11, 20–23]. Moreover, male adolescents are prone to violent injuries because of prevalent risk behaviors such as competitive sports and alcohol consumption [16, 24]. They also tend to use more violent methods, than their female peers, when harming themselves deliberately [11, 12, 23, 25, 26], although there were reports less conclusive [27–29].

In spite of interesting findings, previous studies on adolescent injury have often been based on data from local hospital records or cross-sectional surveys, lacked the details on types of injury, or focused on either accidental or intentional injuries alone [1]. Moreover, no large-scale study, to our awareness, has examined explicitly whether adolescents with self-poisoning are also prone to other forms of injuries, either prior or subsequent to the poisoning. In order to address this gap in the literature, we examined medical records of all external injuries treated in emergency and hospital services among adolescents aged 10–19 years who were treated for medication poisoning in Norway. Our specific objectives are: (1) to examine injuries being treated prior and subsequent to the index medication poisoning by form of injuries and comorbid diagnoses of deliberate self-harm and psychiatric illness, (2) to profile gender differences in specific forms of injuries, and (3) to explore possible associations of prior and subsequent injury incidents with comorbidities of deliberate self-harm and psychiatric disorder diagnosed at the index poisoning. Our hypothesis is that adolescents with a history of medication poisoning are prone to injuries of other forms.

## Methods

### Study population

The study was based on all adolescents aged 10–19 years in Norway. Subjects of focus were adolescents who were identified from the Norwegian Patient Registry (NPR) as having received acute treatment for self-poisoning with medication from 2008 through 2011. The Norwegian version of the Tenth Revision of the International Statistical Classification of Diseases (ICD-10) has been used for medical diagnoses in the NPR. Identification of adolescents into study cohort was carried out in accordance with a previous study focusing on poisoning with medications [8]. Briefly, adolescents who were admitted with a primary diagnosis of poisoning by therapeutic medicaments and biological substances, coded as T4n or T50 in the Norwegian classification system, were included into the cohort and the date of their first poisoning was regarded as the index date in the present study. For this cohort of adolescents, we retrieved incident records for external injuries of all forms (S00-T65) both prior and subsequent to the first recorded treatment because of poisoning with medication, i.e., the index poisoning in the present study. Records of injuries that occurred on the same day or the day after the previous incident were excluded in order to avoid possible duplicate reports of the same incident.

### Study variables

Variables of interest included all external injuries (S00-T65) that led to medical treatment in emergency and hospital services before and after the index medication poisoning. Injury diagnoses were further categorized by specific body regions according to the ICD-10 system as shown in the Tables 1, 2, 3. Injuries with a code of S60 (Superficial injury of wrist and hand) and S61 (Open wound of wrist and hand) were identified because of high relevance to self-cutting behavior. Poisonings by non-medicational substances (T51-T65) and subsequent poisoning with medication (T4n-T50) were considered separately.

**Table 1:** Characteristics of injuries before and after the index poisoning with medication, a gender comparison

Characteristics	Total *N* = 1497 n (%)	Female *N* = 1144 n (%)	Male *N* = 353 n (%)	Female/Male OR^a^ (95% CI)
Any type of injury or poisoning (Yes)				
Before the index poisoning	431 (28.8)	312 (27.3)	119 (33.7)	0.7 (0.6–1.0)
After the index poisoning	586 (39.1)	457 (39.9)	129 (36.5)	NS*
Injuries to the head, neck, and throat (S00-S29) (Yes)				
Before the index poisoning	107 (7.2)	72 (6.3)	35 (10.0)	0.6 (0.4–0.9)
After the index poisoning	119 (8.0)	82 (7.2)	37 (10.5)	0.7 (0.4–1.0)
Injuries to the abdomen, lower back, lumbar spine and pelvis (S30-S39) (Yes)				
Before the index poisoning	214 (14.3)	146 (12.8)	68 (19.3)	0.6 (0.5–0.8)
After the index poisoning	224 (15.0)	172 (15.0)	52 (14.7)	NS
Injuries to the shoulder, arm and elbow (S40-S59) (Yes)				
Before the index poisoning	105 (7.0)	83 (7.3)	22 (6.2)	NS
After the index poisoning	117 (7.8)	105 (9.2)	12 (3.4)	2.9 (1. 6–5.3)
Injuries to the wrist and hand (S60-S69) (Yes)				
Before the index poisoning	133 (10.2)	83 (7.3)	50 (14.2)	0.5 (0.3–0.7)
After the index poisoning	139 (9.3)	97 (8.5)	42 (11.9)	NS
Superficial and open wound injuries to the wrist and hand (S60-S61) (Yes)				
Before the index poisoning	63 (4.2)	38 (3.3)	25 (7.1)	0.5 (0.3–0.8)
After the index poisoning	83 (5.5)	61 (5.3)	22 (6.2)	NS
Injuries to the hip, leg, and foot (S70-S99) (Yes)				
Before the index poisoning	144 (9.6)	106 (9.3)	38 (10.8)	NS
After the index poisoning	146 (9.8)	111 (7.4)	35 (9.9)	NS
Burns, corrosions, and frostbite (T20-T35) (Yes)				
Before the index poisoning	9 (0.6)	7 (0.6)	2 (0.6)	NS
After the index poisoning	10 (0.7)	9 (0.8)	1 (0.3)	NS
Poisonings by non-medicational substances (T51-T65) (Yes)				
Before the index poisoning	73 (4.9)	60 (5.2)	13 (3.7)	NS
After the index poisoning	32 (2.1)	25 (2.2)	7 (2.0)	NS
Poisoning by medications and biological substances (T40-T50) (Yes)				
After the index poisoning	317 (21.2)	274 (24.0)	43 (12.2)	2.3 (1.6–3.2)
Comorbid diagnosis of deliberate self-harm (X6n) (Yes)				
Before the index poisoning	22 (1.5)	18 (1.6)	4 (1.1)	NS
After the index poisoning	184 (12.3)	166 (14.5)	18 (5.1)	3.2 (1.9–5.2)
Comorbid diagnosis of psychiatric disorder (F) (Yes)				
Before the index poisoning	23 (1.5)	14 (1.2)	9 (2.6)	NS
After the index poisoning	189 (12.6)	162 (14.2)	27 (7.7)	2.0 (1.3–3.1)

^a^The analysis was conducted separately for each specific group of injuries; *NS* Not Significant

**Table 2:** Influence of comorbid diagnoses of deliberate self-harm and psychiatric disorder at the index poisoning

Subject Group	Deliberate Self-harm at Index Poisoning			Psychiatric Disorders at Index Poisoning		
	Yes n (%)	No n (%)	OR^a^ (95% CI)	Yes n (%)	No n (%)	OR^†^ (95% CI)
All Subjects: (N = 1497)	668	829		508	989	
Gender						
Female	548 (82.0)	596 (71.9)	1.8 (1.4–2.3)	387 (76.2)	757 (76.5)	NS
Male	120 (18.0)	233 (29.1)		121 (23.8)	232 (23.5)	
Any type of injury or poisoning (S00-T65) (Yes)						
Before the index poisoning	203 (30.4)	228 (27.5)	NS	170 (33.5)	261 (26.4)	1.4 (1.1–1.8)
After the index poisoning	271 (40.6)	315 (38.0)	NS	219 (43.1)	367 (37.1)	1.3 (1.0–1.6)
Injuries to the wrist and hand (S60-S69) (Yes)						
Before the index poisoning	61 (9.1)	72 (8.7)	NS	60 (11.8)	73 (7.4)	1.7 (1.2–2.4)
After the index poisoning	60 (9.0)	79 (9.5)	NS	50 (9.8)	89 (9.0)	NS
Poisoning by medications or other substances (T4n-T65) (Yes)						
Before the index poisoning	27 (4.0)	46 (5.6)	NS	31 (6.1)	42 (4.3)	NS
After the index poisoning	166 (24.9)	163 (19.7)	1.4 (1.1–1.7)	142 (28.0)	187 (18.9)	1.7 (1.3–2.1)
Other injuries (Yes)						
Before the index poisoning	154 (23.1)	163 (19.7)	NS	115 (22.6)	202 (20.4)	NS
After the index poisoning	134 (20.1)	191 (23.0)	NS	100 (19.7)	225 (22.8)	NS
Deliberate self-harm (X6n) (Yes)						
Before the index poisoning	13 (2.0)	9 (1.1)	NS	13 (2.6)	9 (0.9)	2.9 (1.2–6.7)
After the index poisoning	120 (18.0)	64 (7.7)	2.6 (1.9–3.6)	87 (17.1)	97 (9.9)	1.9 (1.4–2.6)
Comorbid psychiatric disorder (F) (Yes)						
Before the index poisoning	11 (1.7)	12 (1.5)	NS	12 (24)	11 (1.1)	NS
After the index poisoning	101 (15.1)	88 (10.6)	1.5 (1.1–2.0)	110 (21.7)	79 (8.0)	3.2 (2.3–4.4)

^a^The analysis was conducted separately for each specific group of injuries; NS = Not significant

**Table 3:** Influences of comorbid diagnoses of deliberate self-harm and psychiatric disorder at the index poisoning in males and females

Subject Group	Deliberate Self-harm at Index Poisoning			Psychiatric Disorder at Index Poisoning		
	Yes n (%)	No n (%)	OR^†^ (95% CI)	Yes n (%)	No n (%)	OR^a^ (95% CI)
**Males** (N = 353)	120 (34.0)	233 (66.0)		121 (34.3)	232 (65.7)	
Any type of injury or poisoning (S00-T65) (Yes)						
Before the index poisoning	48 (40.0)	71 (30.5)	NS	44 (36.4)	75 (32.3)	NS
After the index poisoning	44 (36.7)	85 (36.5)	NS	49 (40.5)	80 (34.5)	NS
Injuries to the wrist and hand (S60-S69) (Yes)						
Before the index poisoning	17 (14.2)	33 (14.2)	NS	23 (19.0)	27 (11.6)	NS
After the index poisoning	14 (11.7)	28 (12.0)	NS	20 (16.5)	22 (9.48)	NS
Poisoning by medications or other substances (T40-T65) (Yes)						
Before the index poisoning	3 (2.5)	10 (4.3)	NS	4 (3.3)	9 (3.9)	NS
After the index poisoning	20 (16.7)	28 (12.0)	NS	22 (18.2)	26 (11.2)	NS
Other injuries (Yes)						
Before the index poisoning	34 (28.3)	47 (20.2)	NS	27 (22.3)	54 (23.3)	NS
After the index poisoning	20 (16.7)	50 (21.5)	NS	18 (14.9)	52 (22.4)	NS
Deliberate self-harm (X6n) (Yes)						
Before the index poisoning	1 (0.8)	3 (1.3)	NS	2 (1.7)	2 (0.9)	NS
After the index poisoning	12 (10.0)	6 (2.6)	4.2 (1.5–11.5)	11 (9.1)	7 (3.0)	3.2 (1.2–8.5)
Comorbid psychiatric disorder (Fn) (Yes)						
Before the index poisoning	2 (1.7)	7 (3.0)	NS	3 (2.5)	6 (2.6)	NS
After the index poisoning	12 (10.0)	15 (6.4)	NS	19 (15.7)	8 (3.5)	5.2 (2.2–12.3)
**Females**(N = 1144)	548 (47.9)	596 (52.1)		387 (33.8)	757 (66.2)	
Any type of injury or poisoning (S00-T65) (Yes)						
Before the index poisoning	155 (28.3)	157 (26.3)	NS	126 (32.6)	186 (24.6)	1.5 (1.1–1.9)
After the index poisoning	227 (41.4)	230 (38.6)	NS	170 (43.9)	287 (37.9)	1.3 (1.0–1.7)
Injuries to the wrist and hand (S60-S69) (Yes)						
Before the index poisoning	44 (8.0)	39 (6.5)	NS	37 (9.6)	46 (6.1)	1.6 (1.0–2.6)
After the index poisoning	46 (8.4)	51 (8.6)	NS	30 (7.8)	67 (8.9)	NS
Poisoning by medications or other substances (T40-T65) (Yes)						
Before the index poisoning	24 (4.4)	36 (6.0)	NS	27 (7.0)	33 (4.4)	NS
After the index poisoning	146 (26.6)	135 (22.7)	NS	120 (31.0)	161 (21.3)	1.7 (1.3–2.2)
Other injuries (Yes)						
Before the index poisoning	120 (21.9)	116 (19.5)	NS	88 (22.7)	148 (19.6)	NS
After the index poisoning	114 (20.8)	141 (23.7)	NS	82 (21.2)	173 (22.9)	NS
Deliberate self-harm (X6n) (Yes)						
Before the index poisoning	12 (2.2)	6 (1.0)	NS	11 (2.8)	7 (0.9)	3.1 (1.2–8.2)
After the index poisoning	108 (19.7)	58 (9.7)	2.3 (1.6–3.2)	76 (19.6)	90 (11.9)	1.8 (1.3–2.5)
Comorbid psychiatric disorder (Fn) (Yes)						
Before the index poisoning	9 (1.6)	5 (0.8)	NS	9 (2.3)	5 (0.7)	3.6 (1.2–10.8)
After the index poisoning	89 (16.2)	73 (12.2)	NS	91 (23.5)	71 (9.4)	3.0 (2.1–4.2)

^a^The analysis was conducted separately for each specific group of injuries; NS = Not significant

Moreover, a supplemental diagnosis confirming intentionality in an injury, coded as X6n in the NPR, was regarded as a recorded deliberate self-harm. We should note, however, data describing the circumstances and the cause of injury were often insufficient in patient records, this diagnosis, i.e. deliberate self-harm (X6n), has been heavily underreported in the patient registry in Norway [30]. Psychiatric comorbidity was defined by having a diagnosis of psychiatric illnesses, coded in F0-F9, either in the primary or secondary diagnoses.

### Statistical analysis

Number of injuries prior and subsequent to the index medication poisoning was counted and distribution by specific types of injury and by gender was explored through descriptive analysis. The associations of injuries with comorbid diagnoses of deliberate self-harm and psychiatric disorder at index poisoning were analyzed. *x*^2^ test was used to detect statistical differences between groups. Odds Ratios (OR) with 95% Confidence Intervals (CI) were estimated through general Logistic regression models. The analysis was conducted separately for each specific group of injuries and moreover by sex. The significance level was set to 0.05 and only tests being statistically significant were reported. All analyses were carried out on SPSS 22.0 or SAS 9.4 software for Windows.

## Results

A total of 1497 adolescents aged 10–19 years received medical treatment because of self-poisoning with medication from 2008 through 2011. These adolescents comprised 1144 (76.4%) girls and 353 (23.6%) boys with a female to male ratio of 3.24. Most of them (89.8%) were between 15 and 19 years old at the time of the index poisoning.

### Exposure to injuries during the study period

Of the 1497 adolescents, 431 (28.8%) individuals were treated at least once for external injuries before the index medication poisoning, and 586 (39.1%) cases had at least one treatment because of injuries after the index poisoning during the observation period. In total, 830 (55.4%) adolescents (55.1% of females and 56.7% of males) received hospital treatment at least on one occasion because of injuries or poisoning either before or after the index poisoning. Altogether 2545 injury incidents, in addition to the index poisoning with medication, were recorded, including 778 injury incidents of other types before the index poisoning and 1767 injuries of all types after the index poisoning.

**Fig. 1:**
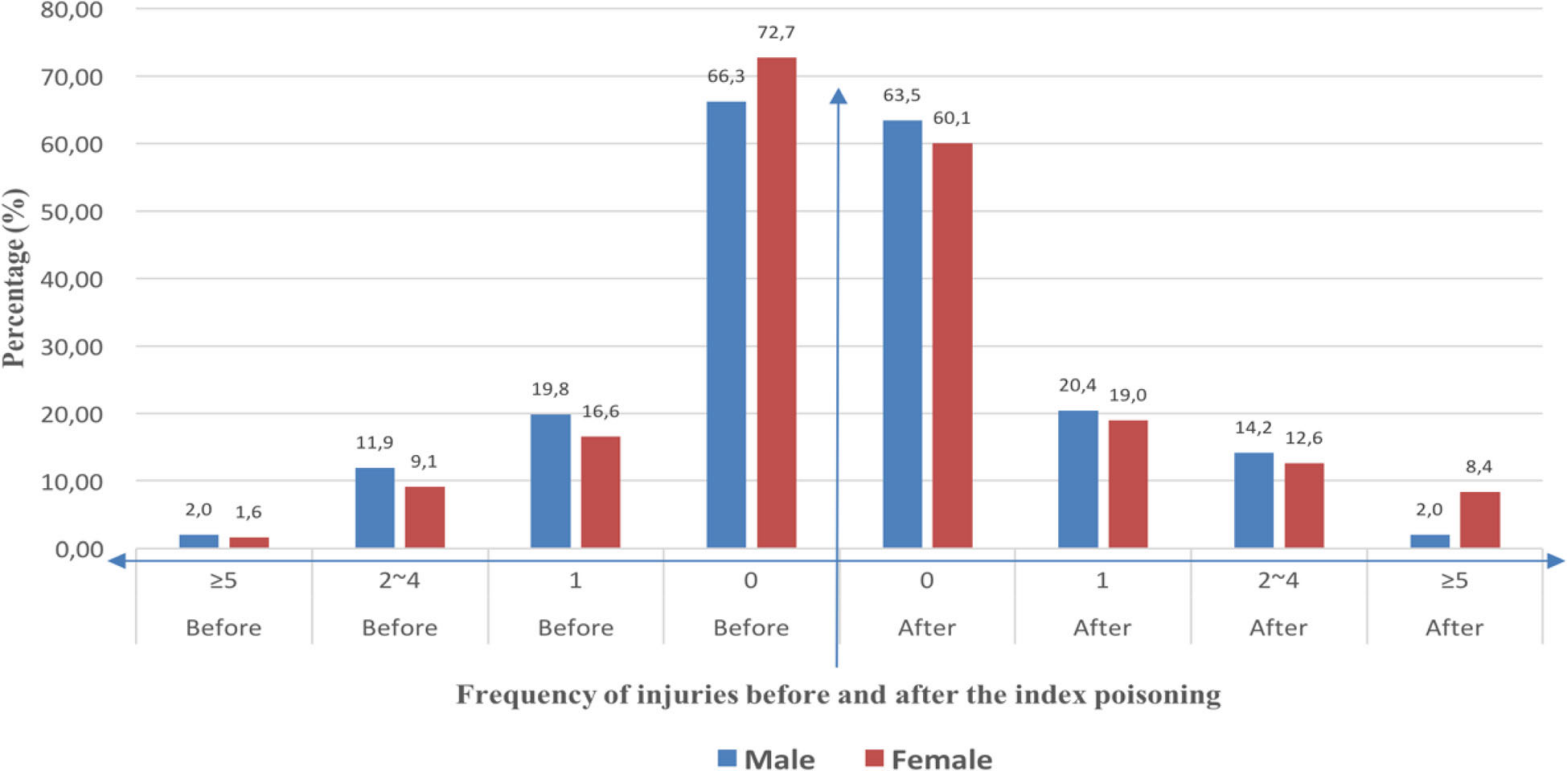
Distribution of injuries before and after the index poisoning with medication

Figure 1 shows the distribution of the number of injuries prior and subsequent to the index poisoning in the study population. Before the index poisoning, one third (33.7%) of male and over a quarter (27.3%) of female adolescents in the cohort had at least one injury treatment, while the corresponding percentages subsequent to the index poisoning were 36.5 and 40.0%, respectively. Many individuals had only one additional injury other than the index poisoning (19.9% of males and 16.6% of females before the index poisoning, and 20.4% males and 19.0% of females after); However, 14.2% of the male and 12.6% of the female subjects had 2 to 4 injury incidents, and 8.4% of females and 2.0% of males had at least 5 incidents subsequent to the index poisoning with medication.

### Form of injuries before and after the index poisoning

The most frequently recorded injury form prior to the index poisoning was injury to the abdomen, lower back, lumbar spine and pelvis (S30-S39), present in 214 (14.3%) individuals and 12.8% of females and 19.3% of males in the cohort (Table 1). The most common form of injury subsequent to the index poisoning was poisoning with medication and biological substances (T4n-T50), present in 317 (21.1%) individuals and 24.0% of females and 12.2% of males, respectively. The second most frequent injury subsequent to index poisoning was injury to the abdomen, lower back, lumbar spine and pelvis (S30-S39), present in 15.0% of all adolescents in the cohort, 15.0% of the females and 14.7% of the males.

### Gender differences in injuries before and after the index poisoning

Table 1 also shows the recorded injuries in male and female adolescents separately. Before the index poisoning, female adolescents had significantly fewer injuries than male adolescents (OR = 0.7, 95% CI: 0.6–1.0). Significant sex differences were seen in certain types of injuries such as injury to the head, neck and throat (S00-S29), injury to the abdomen, lower back, lumbar spine and pelvis (S30-S39), and injury to the wrist and hand (S60-S69), all with a male predominance. Sixty-three (4.2%) individuals had superficial or open wound injuries to the wrist and hand (S60-S61) before the index poisoning; and males were significantly more likely than females to have this type of injury (female:male OR = 0.5, 95% CI: 0.3–0.8). At the same time, females were over twice likely to repeat poisoning with medication (T4n-T50) (24.0% vs. 12.2%; OR = 2.3, 95% CI: 1.6–3.2) after the index poisoning. They were also almost three times more likely to have subsequent injures to the shoulder, arm and elbow (S40-S59) (OR = 2.9, 95% CI: 1.6–5.3).

Moreover, female adolescents were more likely than the males to receive a supplemental diagnosis of deliberate self-harm (OR = 3.2, 95% CI: 1.9–5.2) and also a comorbid diagnosis of psychiatric disorder (OR = 2.0, 95% CI: 1.3–3.1) in connection with their subsequent injuries within the observation period.

### Influence of a recorded deliberate self-harm and psychiatric disorders at index poisoning

At the index poisoning with medication, 668 (44.6%) adolescents received a supplementary diagnosis of deliberate self-harm with a significantly higher presentation in females than males (OR = 1.8, 95% CI: 1.4–2.3). 508 (33.9%) individuals received a comorbid diagnosis of psychiatric disorder with no significant gender difference.

Table 2 presents the injury history of all individuals and Table 3 separates males and females according to whether they were diagnosed with deliberate self-harm or psychiatric disorder at the index poisoning. When both sexes were included (Table 2), a recorded diagnosis of deliberate self-harm at the index poisoning increased the likelihood of receiving a supplementary diagnosis of deliberate self-harm (OR = 2.6, 95% CI: 1.9–3.6), as well as a comorbid psychiatric diagnosis at subsequent injuries (OR = 1.5, 95% CI: 1.1–2.0). Furthermore, such a diagnosis was also significantly associated with an increased risk of having subsequent poisoning incidents (OR = 1.4, 95% CI: 1.1–1.7).

Individuals diagnosed with a psychiatric comorbidity at index poisoning had significantly more injuries both prior and subsequent to index poisoning (OR = 1.4, 95% CI: 1.1–1.8 (before); OR = 1.3, 95% CI: 1.0–1.6 (after)). These adolescents were also more likely to have injuries to wrist and hand before the index poisoning (OR = 1.7, 95% CI: 1.2–2.4 for S60-S69; OR = 2.1, 95% CI: 1.3–3.5 for S60-S61), poisoning with medication after the index poisoning (OR = 1.7, 95% CI: 1.3–2.1), and to receive a diagnosis of deliberate self-harm both before (OR = 2.9, 95% CI: 1.2–6.7) and after (OR = 1.9, 95% CI: 1.4–2.6) index poisoning. Individuals receiving a comorbid psychiatric diagnosis at index poisoning were three times more likely to receive a psychiatric diagnosis at subsequent injuries (OR = 3.2, 95% CI: 2.3–4.4).

When males and females were analyzed separately (Table 3), a supplementary diagnosis of deliberate self-harm at the index poisoning was positively associated with a diagnosis of deliberate self-harm given in subsequent injuries in both sexes (OR = 4.2, 95% CI: 1.5–11.5 for males and OR = 2.3, 95% CI: 1.6–3.2 for females). A comorbid diagnosis of psychiatric disorder at index poisoning was positively associated with the presence of comorbid diagnoses of deliberate self-harm (OR = 3.2, 95% CI: 1.2–8.5) and psychiatric disorder (OR = 5.2, 95% CI: 2.2–12.3) at subsequent injuries among male adolescents.

Female adolescents who received a comorbid diagnosis of psychiatric disorders at index poisoning were more likely to receive treatment for both prior (OR = 1.5, 95% CI: 1.1–1.9) and subsequent (OR = 1.3, 95% CI: 1.0–1.7) injuries of any form. Specifically, they more often had prior injuries to the wrist and hand (S60-S69) (OR = 1.6, 95% CI: 1.0–2.6) and subsequent poisoning with medication and other substances (T40-T65) (OR = 1.7, 95% CI: 1.3–2.2). More notably, these females were most likely to get a supplemental diagnosis of deliberate self-harm (OR = 3.1, 95% CI: 1.2–8.2 (before); OR = 1.8, 95% CI: 1.3–2.5(after)) as well as a comorbid diagnosis of psychiatric disorder at injuries both before and after the index poisoning (OR = 3.6, 95% CI: 1.2–10.8 (before); OR = 3.0, 95% CI: 2.1–4.2 (after)).

## Discussion

To our knowledge, this is the first population study examining external injuries of various forms in a cohort of adolescents treated for self-poisoning with medication using data from a national patient registry. The results support our hypothesis indicating that: 1) external injury among the cohort adolescents was quite common and manifested itself in a variety of injuries, 2) the presence of both prior and subsequent injuries was highly associated with a comorbid diagnosis of deliberate self-harm or psychiatric illness at index poisoning, and that 3) there were significant gender differences in injury incidents and form of injuries.

### Commonness of injuries of other forms

External injuries were prevalent in our cohort of adolescents, with over half of them having at least one injury before or after the index poisoning. About 30% of these adolescents had a record of treatment because of injuries in forms other than medication poisoning before the index poisoning – an insightful detail which has not been reported in related studies. In the meantime, nearly 40% of the cohort adolescents were treated for injuries of various forms after the index incident of medication poisoning.

The commonness of injuries of other forms, seen in our cohort both before and after the index poisoning, supports the notion in previous studies [12] that methods of injury change during repeat episodes of self-harm. Our data showed that injury to hand and wrist was rather common, presenting in over 10% of the adolescents prior to the index poisoning and 9.3% of them subsequently to the index poisoning. This is probably an underestimation of the true number of such injuries since we have only analyzed hospital treated injuries. Poisoning, on the other hand, was the most frequent injury subsequent to the index poisoning.

Our finding that less than half of the adolescents in the cohort received a comorbid diagnosis of deliberate self-harm at their index poisoning was clearly an underreporting of this diagnosis in the patient registry, as compared with the rates reported by studies [30–33] in which the intentionality of poisoning was additionally reviewed. Similarly, the 33.9% psychiatric comorbidity found in the present study is much lower than the report that approximately 8 in 10 adolescents who self-harm have psychiatric disorders [34]. Despite of possible underreporting, the relatively higher rates of deliberate self-harm and psychiatric disorders at index poisoning in females than males are consistent with the findings from previous research [2, 31]. The observed strong associations of deliberate self-harm and psychiatric comorbidity being diagnosed at index poisoning with both prior and subsequent injuries of other forms are also in high concordance with studies on self-harm repetition among adolescents [3, 11, 12, 35–38]. These findings underscore the importance of assessment of mental status and intentionality for adolescents presenting to health services for treatment because of poisoning and injury.

### Specific forms of injuries by gender

The present study did not detect a highly distinct pattern of injuries between males and females in the cohort, but several findings separated the genders.

Common forms of injury observed in males, both before and after the index poisoning, was injury to the abdomen, lower back, lumbar spine and pelvis (S30-S39) and injury to the head, neck and throat (S00-S29). Females on the other hand showed predominance of injury to the shoulder, arm and elbow (S40-S59). We cannot ascertain, however, whether this illustrates a difference in preferred method of self-injury or simply reflects gender differences in forms of non-intentional injuries likely happening in daily life.

Interestingly, males in the cohort stood out as having significantly more injuries to the hand and wrist prior to the index poisoning. Community studies on self-cutting suggest that this behaviour was more frequent in females than males [29, 39], whereas a male predominance was reported in hospital treated injuries to the hand and wrist [40, 41]. It is therefore possible that females may be overrepresented in hand and wrist injuries not treated in hospital services (community studies), while males are overrepresented in such injuries presenting to hospital [40]. A possible explanation to this could be that injuries of self-cutting on hands and wrists in female adolescents are less often severe and thus go untreated but in male adolescents are more often severe and thus are treated.

Female adolescents diagnosed with a psychiatric disorder at index poisoning seemed to be particularly vulnerable to having sustained injuries both before and after the index poisoning and to receiving a supplemental diagnosis of deliberate self-harm (X6n) on these occasions. They were more often treated for injuries to the wrist and hand before the index poisoning and for repeat poisoning after the index poisoning. These findings support the argument of a possible switch of methods used for self-harm in this group of adolescents [12, 40, 42].

Males in our cohort were significantly more likely than females to have a prior injury to the wrist and hand (S60-S61) for which no comorbid diagnosis of deliberate self-harm (X6n) was given. This is an interesting finding given the high likelihood of such injury being a deliberate action. Females, on the other hand, were more likely to repeat poisoning with medication and also to receive comorbid diagnoses of deliberate self-harm and psychiatric disorder at their subsequent injuries. A possible reason for this could be that adolescents, especially boys, often attend sport activities, so their injuries on hand and wrist are commonly perceived as accidental instead of deliberate. Another possible explanation for the discrepancy might be that, from a clinical point of view, poisoning with medication occurs more often with intention as compared to injury to the hand or wrist (having in mind that these injuries can be superficial). This is in line with previous research [40, 43] stating that suicidal intent is higher in individuals who self-poison compared to individuals who self-injure. Regardless of the reason, the low rate of deliberate self-harm being given to injuries to the hand and wrist might constitute a problem, because self-injury, particularly self-cutting, is associated with not only risk of repetition, but also risk of suicide completion in adolescents, particularly in males [6, 40, 42]. In the meantime, the higher rate of psychiatric diagnoses for female compared to male adolescents following index poisoning may imply an under-reporting by males of psychological distress and, consequently, an under-treatment of male mental ill-health.

### Limitations and strengths

The data obtained for the present study ensured the accuracy of episodic hospital contacts because of injury, but did not contain personal profiles such as socioeconomic status, detailed information on psychiatric diagnosis and other health related records that may have provided interesting insights. Also, for injuries treated in emergency clinics, the assessment of psychiatric illness and deliberate self-harm was not systematically carried out. This may impose an underestimation of the true prevalence of such comorbidities among injury incidents and limit the possibility to fully distinguish between self-inflicted injuries and injuries inflicted by others. Furthermore, since many mild injuries do not lead to medical care, this study could not generalize to those who do not present to emergency or hospital services after injury and does in no way reflect the incidence and frequency of injuries or deliberate self-harm among adolescents. In addition, data on type of medication used in self-poisoning was not available. It would have been of great value to identify whether the medications were prescribed or purchased over the count so that to inform strategies of control to prevent future self-poisoning.

Despite of these limitations, the present study has several strengths. It is, to our knowledge, the first one that has examined a large cohort of adolescents with medication poisoning for their external injuries of various forms. Unlike studies relying on self-reports and clinical records, this study is based on precise data of patient records with a standardized diagnostic classification of injuries and comorbidities. The longitudinal nature of the data has enabled the possibility of looking into the details both retrospectively and prospectively.

## Conclusion

This study shows that adolescents with medication poisoning represent a high-risk group prone to both prior and subsequent injuries of various forms in need of hospital treatment. Possible switches in injury methods as well as gender differences in injury incidents and forms were observed. A male predominance of injuries to hand and wrist was found together with an increased likelihood of subsequent poisoning in females. Comorbid diagnoses of deliberate self-harm and psychiatric illness at index poisoning correlated strongly with prior as well as subsequent injuries in the cohort adolescents, especially among the females. With growing evidence for a positive effect of psychosocial assessment for patients treated for self-harm on repetition reduction [7], the present study further pinpoints that evaluation of mental health status and intentionality should be implemented to the utmost extent among adolescents presenting to health services because of self-poisoning or injuries.

## Abbreviations

CI: Confidence Intervals
ICD-10: the Tenth Revision of the International Statistical Classification of Diseases
NPR: Norwegian Patient Registry
NS: Not significant
OR: Odds Ratio
